# Fisheye-Based Smart Control System for Autonomous UAV Operation

**DOI:** 10.3390/s20247321

**Published:** 2020-12-20

**Authors:** Donggeun Oh, Junghee Han

**Affiliations:** School of Electronics and Information Engineering, Korea Aerospace University, 76 Hanggongdaehang-ro, Goyang-si, Gyeonggi-do 412-791, Korea; gon023415@kau.kr

**Keywords:** IoTs, UAVs, machine-learning, autonomous flight, VIN, Fisheye

## Abstract

Recently, as UAVs (unmanned aerial vehicles) have become smaller and higher-performance, they play a very important role in the Internet of Things (IoT). Especially, UAVs are currently used not only in military fields but also in various private sectors such as IT, agriculture, logistics, construction, etc. The range is further expected to increase. Drone-related techniques need to evolve along with this change. In particular, there is a need for the development of an autonomous system in which a drone can determine and accomplish its mission even in the absence of remote control from a GCS (Ground Control Station). Responding to such requirements, there have been various studies and algorithms developed for autonomous flight systems. Especially, many ML-based (Machine-Learning-based) methods have been proposed for autonomous path finding. Unlike other studies, the proposed mechanism could enable autonomous drone path finding over a large target area without size limitations, one of the challenges of ML-based autonomous flight or driving in the real world. Specifically, we devised Multi-Layer HVIN (Hierarchical VIN) methods that increase the area applicable to autonomous flight by overlaying multiple layers. To further improve this, we developed Fisheye HVIN, which applied an adaptive map compression ratio according to the drone’s location. We also built an autonomous flight training and verification platform. Through the proposed simulation platform, it is possible to train ML-based path planning algorithms in a realistic environment that takes into account the physical characteristics of UAV movements.

## 1. Introduction

Recently, UAVs are becoming smaller and more intelligent, and UAVs play a very important role in the Internet of Things (IoTs). Hence, their fields of use are expanding to various fields [1,2]. In the past, they were developed and used for military purposes, but, nowadays they are used in various environments such as agriculture, distribution, logistics, and construction. For example, a German international express delivery company, DHL, is working on a project to deliver drugs with drones. In addition, Alibaba in China is developing drones for logistics delivery and plans to use them in mountainous and island areas in the future [3,4]. Furthermore, many UAVs are being deployed in mission-critical services, such as tracking wide disaster sites and delivering emergency kits for rescue mission scenarios [5].

The increasing demand for UAVs has led to the need for development of sophisticated and intelligent drone software. In particular, there is a need for the development of an autonomous system in which a drone can determine and accomplish its mission even in the absence of remote control from a GCS (Ground Control Station). Especially, it is very important for drones to perform their duties safely without colliding with obstacles such as buildings and terrain even if they are not controlled by humans. Responding to the needs, there have been various studies and algorithms developed for autonomous flight systems. Especially, many ML-based (Machine-learning based) methods have been proposed for autonomous path finding [6,7,8,9,10,11,12,13,14,15,16,17,18,19,20,21,22,23]. However, they are limited when applied to a large target area. Furthermore, they perform poorly in a new environment different from the trained and learned environment.

To address this issue, this paper proposes a drone autonomous tracking system for sustainable flight operations even in a wide target area. The main contributions of this paper can be summarized as follows.

- We develop an effective and efficient deep learning-based path planning algorithm. Compared to previous ML-based path planning algorithms, the proposed technique can be applied to a wide target area without sacrificing accuracy and speed. Specifically, we propose a Fisheye hierarchical VIN (Value Iteration Networks) algorithm that applies different map compression levels depending on the location of the drone.
- We build an autonomous flight training and verification platform. Through the proposed simulation platform, it is possible to train ML-based path planning algorithms in a realistic environment that takes into account the physical characteristics of UAV movements. Moreover, thanks to the platform, the proposed autonomous flight algorithm can be verified in a realistic and practical way.

The rest of the paper is organized as follows. Section 2 introduces existing path planning algorithms and simulation platforms and discusses the problems and limitations. Section 3 proposes the Fisheye HVIN and Multi-Layer HVIN path planning algorithms for wide-area autonomous flight systems. In Section 4, we present the design of an integrated training and simulation platform, describe the experimental procedure, and analyze the results for the performance evaluation of the proposed approach. Finally, Section 5 wraps up this paper with discussion.

## 2. Background 

### 2.1. Path Planning

Path planning to the target point is the most basic and important process in completing an autonomous flight mission [24,25]. There have been many research studies for efficient and effective path planning. One of the representative and well-known path planning algorithms is *A star* Algorithm (referred as $A^{*}$) [26,27]. $A^{*}$ is an algorithm that plans the shortest path to the target point based on a heuristic function. Specifically, this algorithm scores each path it explores before and continues this search process. By storing the route passed through the previous steps, the agent can search the shortest route from the starting point to the destination. However, in autonomous flight scenarios, it is common and frequent that the map changes or the size of the target area increases after determining the shortest route. However, $A^{*}$ has difficulty responding flexibly to these network changes. This is because it is inefficient and impractical for $A^{*}$ to compute heuristic values every time the network changes.

**Figure 1:**
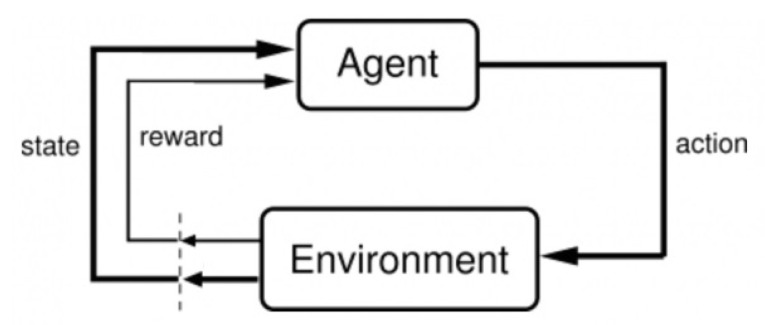
A schematic diagram of the reinforcement learning process.

Alternatively, several ML-based approaches have been proposed for path planning. DQN (Deep Q Networks) [8,9,10] is one of the well known and widely used ML-based path planning algorithms. Basically, DQN is categorized as a reinforcement learning method, which learns how to make the best decision in the future through the process of performing an action and receiving a reward [28,29,30]. Figure 1 presents a schematic diagram of the reinforcement learning process. Agents learn in the environment of the current state and make decisions. The environment changes according to the action and determines the reward. DQN intends to choose the action with the greatest overall reward expected in the future. The expected reward according to the action (*Q*-value) is obtained through the Q function. At this time, a rule for selecting an action in a specific state is called a policy. Step-by-step details of the algorithm can be found in the work of Mnih et al. [8].

**Figure 2:**
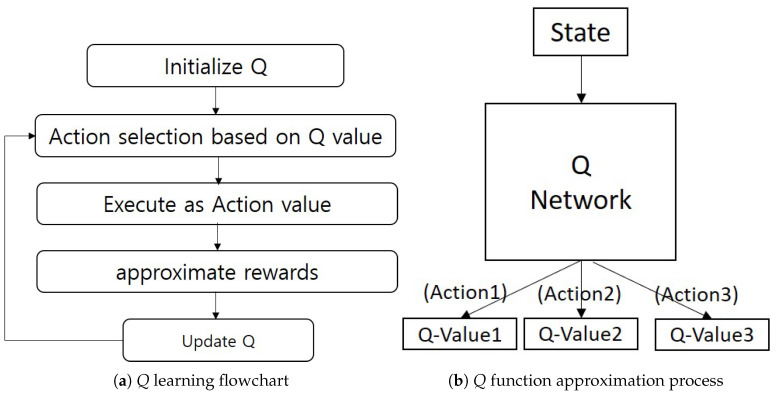
*Q* learning process.

The DQN algorithm approximates the *Q* value function by repeating the action according to the policy or action value and repeating the process of obtaining rewards, as shown on the left of Figure 2. Specifically, *Q* neural network is used for approximation of the *Q* value function. This process is illustrated on the right of of Figure 2. The *Q* network receives a state (e.g., image information of Atari game DQN [30]) and calculates the *Q*-value for each action in the action space.

The update of *Q*-value is executed using an optimizer. Because an optimizer is used, the loss function, that is, the objective function, must be defined. The loss function can be simply defined as the squared error of the target value of *Q*-value and the prediction value as in Equation (1).

(1) $$Loss=1/2*\{r+max_{a^{\prime }}Q\left(s^{\prime },a^{\prime }\right)-Q\left(s,a\right)\}$$

By using state *s* as an input, Q-network forward pass is performed to obtain action values for all actions. After getting $<r,s^{\prime }>$, which is the return value of environment for action *a*, state $s^{\prime }$ is used again to get action values for all action $a^{\prime }$. Then, we get all the information to find the above loss function. The loss function updates the weight parameter so that the *Q*-value update for the selected action converges, that is, the target value and the predicted value become as close as possible.

Based on DQN, various extensions and applications have been developed. To enhance the original DQN, Han [11] developed Double DQN, which exploited a priority sample replay in a 3D obstacle avoidance environment. Kjell [12] compared DQN, Double DQN, and Dueling DQN methods. In addition, A3C (Asynchronous Advance Actor–Critic) [31] is proposed, especially for many gaming applications [32]. Unlike DQN, A3C uses two networks: actor network and critic network. However, A3C is still impractical for taking braking action and changing speed on a binary level consisting of two separate values. Furthermore, these DQN and DQN-extended algorithms are mainly for discrete actions.

For realistic driving scenarios, DDPG (Deep Deterministic Policy Gradient) [13] adapts the ideas of DQN to the continuous action domain. Many extended studies of DDPG have been developed for various applications [14,15,16,17]. Kong et al. [14] used state-adversarial deep deterministic policy gradient algorithm (SA-DDPG) for combat maneuver decisions of an opponent aircraft being considered assuming gun-based aerial combat WVR. Gupta et al. [15] proposed an environment perception framework for autonomous driving using state representation learning (SRL). Unlike existing Q-learning based methods, Gupta et al. [15] took the learning loss into account under deterministic as well as stochastic policy gradient by combining Variation Autoencoder (VAE), Deep Deterministic Policy Gradient (DDPG), and Soft Actor–Critic (SAC). Qi et al. [16] presented a UAV control policy based on DDPG to address the combination problem of 3D mobility of multiple UAVs and energy replenishment scheduling. In addition, a MEP–DDPG algorithm was designed using model predictive control and simulated annealing to generate expert experiences [17]. The authors proposed a MEP–DDPG algorithm to address UAV’s AMP (Autonomous Motion Planning) problem. The authors of [18,19] proposed an autonomous landing task mechanism of UAVs based on sequential DQN and DDPG algorithms, respectively.

However, DQN- and DDPG-based algorithms have several limitations to be applied to an autonomous flight system. The main limitation is that these algorithms perform poorly especially in a new environment that is very different from the trained area a priori. It also takes long time to be trained in the large area networks similar to non ML-based algorithms such as $A^{*}$.

Compared to the previous methods, VIN (Value Iteration Networks) allows an agent to learn planning to reach a target even in a new environment [20,21]. VIN is also one of the reinforcement learning models similar to DQN, but it additionally has embedded separate explicit reactive planning modules to express the policy.

In particular, it receives information about the map (grid-world), goal, and position of a drone as input values to the algorithm. Based on these input data, the reward value from the CNN process is calculated. The value iteration process takes the highest value closest to the current state and propagates this value. After that, it deduces approximation value for action and learns reactive-policy through attenuation process. In this procedure, unlike DQN, VIN contains a planning module that learns to plan from model-free objectives given by a value function. In other words, VIN is to create an explicit NN-based reactive policy that can learn planning, which is called a value-iteration network. A *“planning program”* that can be differentiated is embedded in the NN. The goal is to generalize the solving mechanism by learning the planning itself. Thanks to this feature, VIN works well for tasks that involve planning-based reasoning (e.g., navigation tasks) from one initial position to one goal position. In addition, VIN shows better performance than other reinforcement planning methods, especially in a new environment.

In addition, Radac and Lala [23] showed that VIN algorithm’s convergence is guaranteed under general function approximators with providing a case study for a low-order linear system in order to generalize the more complex ORM tracking validation on a real-world nonlinear multi-variable aerodynamic process. Considering these advantages of VIN over DQN- or DDPG-based algorithms, we choose a VIN method as a baseline path planning algorithm. Note that, however, the original VIN has a limitation of state space (e.g., 16 × 16 grid), which makes it difficult to apply to large area autonomous tracking systems. To make up for the limitations, this paper proposes *“hierarchical”* VINs, as explained in Section 3.

### 2.2. Simulation Platform

In order for autonomous flight path finding to be practical, algorithms and technologies must be verified through simulation in a realistic environment. Thus far, many robot simulators have been developed such as Gazebo and Vrep with which we can simulate the physical movement of UAVs [33,34,35]. Gazebo [33] is a widely tool used for the development of robots with various physical characteristics, such as conveyor belts, unmanned probes, and line tracers. This tool can incorporate various sensor modules for these robots, and thus we can make a UAV equipped with Wi-Fi signal detection sensor or a camera sensor. Vrep [34] is also a robot simulator with an integrated development environment based on a distributed control architecture. Each object and model can be individually controlled through embedded scripts, plug-ins, ROS or BlueZero nodes, remote API clients, or customization. Controllers can be written in C/C ++, Python, Java, Lua, Matlab, or Octave. Based on these characteristics, Vrep is widely used for rapid algorithm development, factory automation simulation, rapid prototyping and verification, robot-related education, remote monitoring, and safety double inspection.

ROS (Robot Operating System) is a software platform for robot control [36,37]. It provides hardware abstraction, sub-device control, sensing or recognition, and message passing between processes required for robot application development. ROS handles a process that performs computation as a node. Each node sends and receives messages to the publisher–subscriber structure through a channel called *Topic* or exchanges messages in a *Service* method. Through these methods, it is possible for a ROS node to exchange information with another ROS node or to share information with a desired node.

In this paper, ROS is used for communication between the proposed reinforcement learning-based path search node in the autonomous tracking system (i.e., HVIN Agent) and the drone modeling node in a Vrep simulator. The node-to-node communication in the proposed platform includes a topic communication method of a publishing and subscription concept as well as a service method that nodes communicate in a request and response procedure.

## 3. Proposed Approach

Although various path planning studies including heuristic- and deep-learning based-algorithms have been developed thus far, applying these algorithms for wide-area autonomous flight system is still a challenging task because they are either not applicable to wide-area systems or not adaptive to a new environment or dynamic circumstances. In what follows, this paper identifies important issues to solve for a wide-area autonomous flight system and proposes two types of HVIN algorithms.

### 3.1. Problem Statement

As mentioned in Section 2, previous path planning studies have common limitations that they are not applicable to cover wide autonomous flight areas. To address this issue, this paper proposes a hierarchical smart drone control system for wide-area autonomous drone flight. The basic idea is that we expand the size of application by dividing the large target area into different levels of layers. Depending on the size of a target and size limits of the original algorithms, the depth of layers (or number of layers) can be more than two.

At first glance, simple hierarchizing or layering the target area is the traditional and straightforward way to extend any given algorithm to cover a wide-area [38,39]. However, with overlaying layers in deeper levels, we might sacrifice accuracy and efficiency with a large volume of overhead. To overcome such limitations and problems of layering, this paper proposes a Fisheye hierarchical VIN algorithm which “hierarchizes” the target area with “adaptive” compression rate. In this HVIN, the area around the a UAV is analyzed in detail, whereas information in other areas far from the current UAV location is greatly compressed and roughly analyzed. That is why we call this method as Fisheye-Based HVIN.

This method adopts VIN [20,21] as a baseline ML-based path planning and dynamically constructs hierarchical VIN layers to cover a wide area.

**Figure 3:**
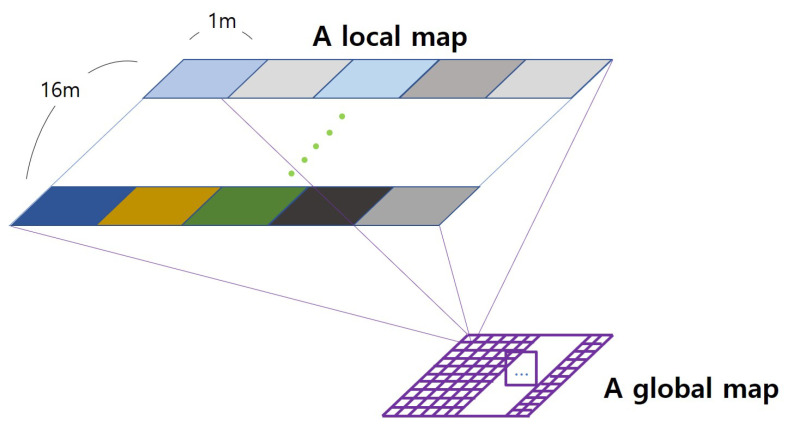
Basic two-layer hierarchical VIN.

To explain the concept of the proposed Fisheye HVIN (Hierarchical VIN), let us assume a hierarchical VIN using two layers, a local layer and a global layer, with the static compression rate for now, as illustrated in Figure 3. Generally, the existing VIN model consists of 16 × 16 grid-world sections [20,21]. Thus, one section out of the 256 sections in a 16 × 16 grid at a global layer corresponds to the whole 16 × 16 area of the local layer. (Note that we assign 1 m to an *edge gap* for boundaries of the local layer map to identify edges of the local map. Because of this, the actual coverage size of the local layer area becomes 14 m × 14 m excluding the edge gap around each local map.) The global layer compresses each local area into one global section of the global map and performs path planning based on the global map data. If we allocate 1 m × 1 m to each section of a local layer, then the total coverage of two-layer HVIN becomes (16 × 16) × (16 × 16) m${}^{2}$.

In the original VIN algorithm, each section is labeled with either 0 if free space or 1 if it contains any obstacle(s). In this paper, the local layer acts as the original VIN and so each section of the local layer is labeled with either 0 or 1. In contrast, each section of the global layer is correspondent to the 16 × 16 local area, which means that one global section contains 256 local sections. Hence, each section of the global layer might include more than one local layer sections with obstacles. To accommodate this situation, we label it with the number between 0 and 256 (=16 × 16), which indicates the number of local layer sections containing obstacles. Additionally, we define *"Obstacle Factor"*. If the label of a global section is lower or higher than the obstacle factor value, each global section is determined as free space or obstacle area, respectively. By adjusting the obstacle factor value, we can customize our algorithm in either a conservative or an aggressive way considering the target application’s feature.

**Figure 4:**
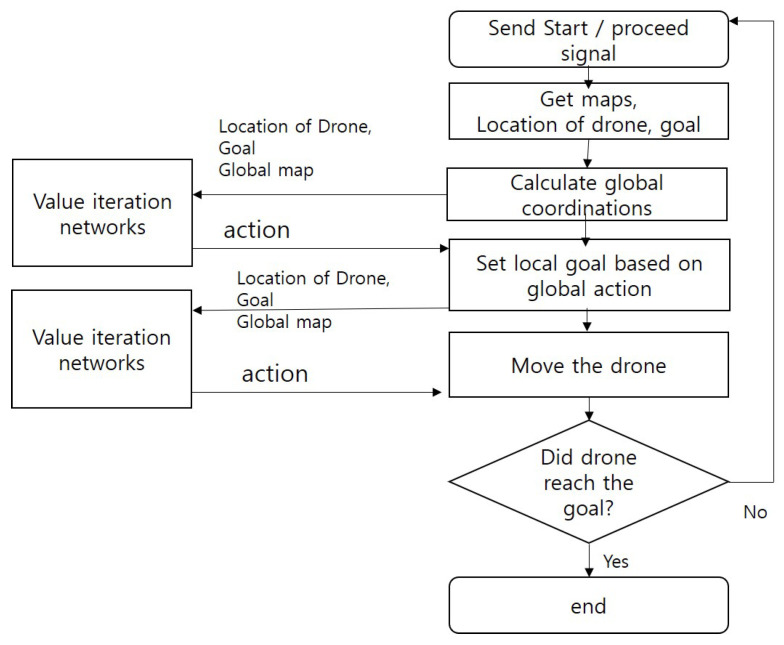
Procedure of Hierarchical VIN.

The detailed operating procedure of a hierarchical VIN (HVIN) algorithm is presented in Figure 4. When a drone is running, a HVIN module receives the data about drone’s position, final destination, and sensed data around the drone from Vrep ROS node. Using the data, the HVIN agent computes the global position for the drone and a local goal point and constructs a global image map using *Obstacle Factor*. Then, a local VIN agent sets a local target point in a local layer based on a planned action. Based on the location of the drone and a local target, it computes and sends a local action to the drone in a Vrep simulator.

The maximum target area of the above example is 256 × 256 because it has only two layers with the fixed compression rate (i.e., mapping 16 × 16 to 1). To further expand this basic HVIN idea, this paper develops Multi-Layer HVIN and Fisheye HVIN as methods to extend the size of autonomous flight area. In what follows, we present the detailed algorithms of the two types of HVIN:

- Multi-Layer HVIN
- Fisheye HVIN

### 3.2. Multi-Layer Hierarchical VIN

**Figure 5:**
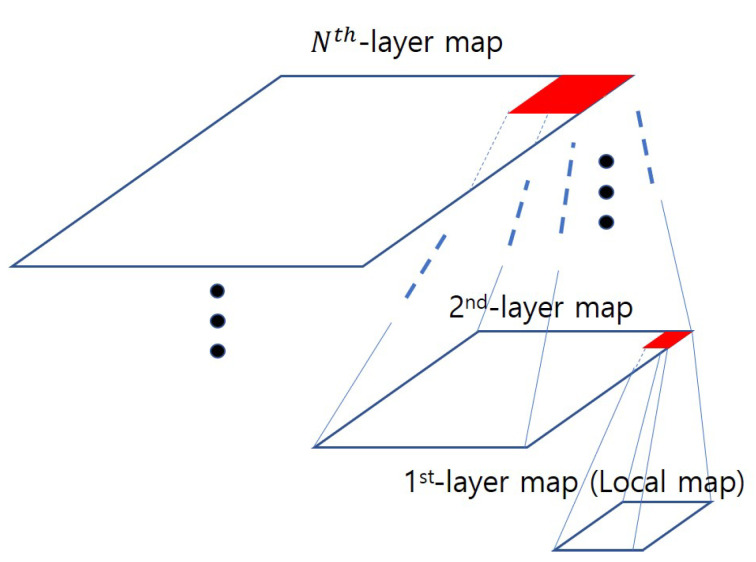
Concept of the Multi-Layer Hierarchical VIN.

We extend the idea of two-layer HVIN described in the previous section to a more general Multi-Layer Hierarchical VIN (MHVIN) (Figure 5), by constructing multiple layers to broaden the range of a target area applicable to autonomous flight. For example, to accommodate 2 km × 2 km area, two layers are not enough and so we need to construct one more layer, i.e., three-layer HVIN. The operation procedure of three-layer HVIN is very similar to the one of two-layer HVIN. We add the operation of the third layer in the procedure of two-layer HVIN as follows. First, the third layer position is calculated based on the coordinates of the drone and the destination. Then, third layer VIN agent computes the action of drone at third map resolution. Then, we determine the waypoint of the drone based on the output in the third hierarchy. The waypoint is considered as a target of the second hierarchy section. When the drone reaches within a certain range of this waypoint, it considers that it reaches the “goal” and sets up a new waypoint again through the third hierarchy. The same procedure is recursively applied between the second layer waypoint and the first layer (i.e., local layer) drone action. It iterates this procedure until it reaches its final destination.

With adding more layers, this method can be applied to a wide-area system. However, it requires more layers as the size of an autonomous flight application area increases. Thereby, the amount of map data to be transmitted and computation overhead is increasing. Furthermore, the recursive operation procedure of multiple HVIN becomes more complicated and even impractical in the real-world.

### 3.3. Fisheye Hierarchical VIN

To complement limitations of Multi-Layer HVIN in the previous section, we develop a **Fisheye Hierarchical VIN** algorithm. The main idea of the proposed Fisheye HVIN is summarized as follows.

- **Adaptive compression rate**: It applies a different compression level to the image map of the global layer according to the location of the drone.
- **No more than two layers**: It keeps the depth of layering up to two layers. Thus, it can avoid recursive operations with a large volume of overheads.
- **Covering unlimited size of area**: It can be applied to the large area without size limitation.

**Figure 6:**
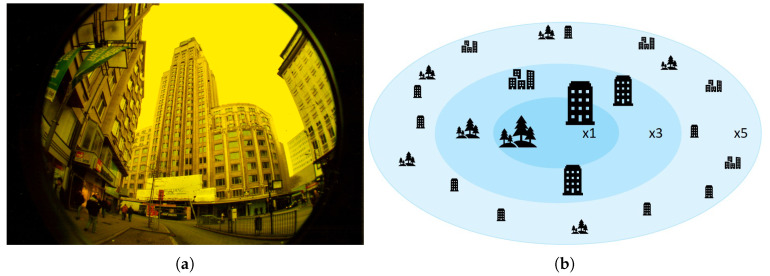
Concept of Fisheye HVIN: (**a**) fisheye view [42]; and (**b**) fisheye diagram.

In this proposed method, we adopt the idea of fisheye lens [40,41], which provides a very wide viewing angle through refraction of light, as shown in Figure 6. As with fisheye lens, this paper considers that the farther sections are from the drone in the global hierarchy, the more compact the sections are. In other words, Fisheye Hierarchical VIN applies less compression to the map as it gets closer to the location of the drone in order to obtain more detailed information around the drone. Conversely, it compresses the map more when the area is farther from the drone.

**Figure 7:**
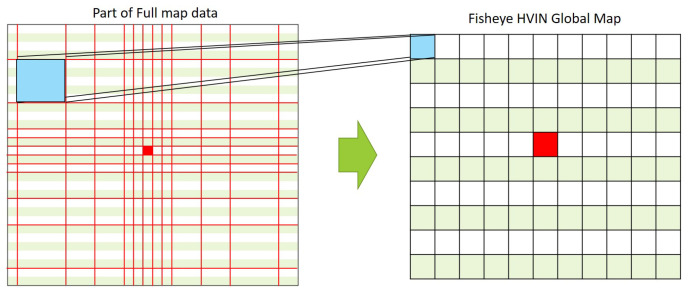
Proposed Fisheye HVIN mapping.

For example, in Figure 7, suppose the red part is the current location of a drone. In this figure, the left map is a real one without any compression whereas the right one is a 16 × 16 global map for the target area. In the entire map data, the local area close to the drone is mapped to the global map without compression, as represented as one red section. In contrast, a larger area far from the drone, for example, represented as a blue square in the left map, is also mapped to only one section in the global map in the right figure. Even though the blue area is larger than the red area in the left map, both are mapped to the same size sections in the right map of Figure 7. This means that the area farther from the drone is more compressed than the closer area.

**Figure 8:**
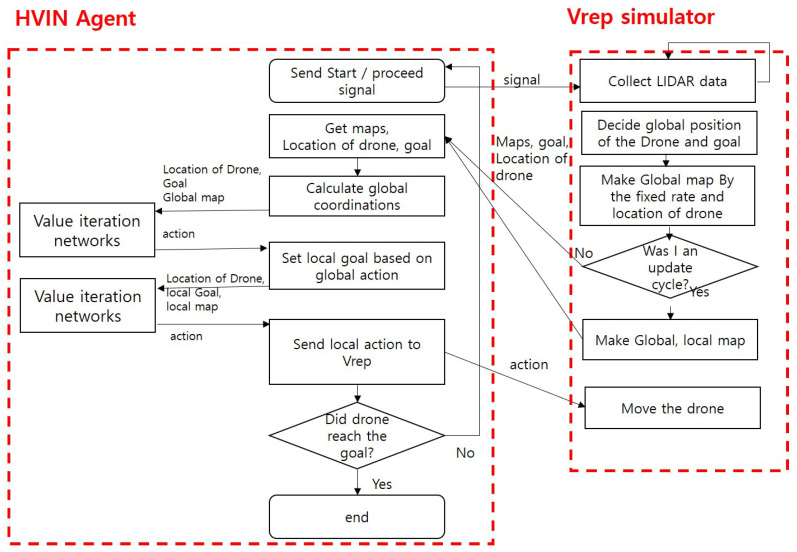
Procedure of Fisheye Hierarchical VIN.

The overall operating procedure of the Fisheye hierarchical VIN in Figure 8 is explained as follows.

1. The simulator calculates the drone position in the global map, taking into account the magnification in the global hierarchy. Specifically, it calculates the drone’s global position by subtracting Nx14 to match the magnification *N* of the *x* and *y* coordinates of the absolute position.
2. It calculates a global position of the goal based on the calculated position of a drone.
3. It calculates the boundary to be divided into a global map considering the magnification *N* based on the absolute position of the drone. It maps the calculated boundary to the global map.
4. A Fisheye HVIN agent receives data from a simulator and performs HVIN operation by running VIN in the global map and running another VIN procedure in the local map.
5. The HVIN agent sends the action value back to the simulator so that the drone can move complying with the action order from the FHVIN agent.

Thanks to these characteristics of Fisheye HVIN, the size of the area applicable to autonomous flight can be flexibly adjusted according to magnification. In addition, the compression level of the global image map varies depending on the location of the drone. Therefore, the simulator such as Vrep in our study should calculate the global map and pass it to Fisheye HVIN every time the drone moves. To reduce the computational complexity on the simulator side, we design a platform that periodically updates the global map instead of computing the global map for every movement of the drone.

## 4. Evaluation

### 4.1. Simulation-Based Training and Verification Platform

Many machine learning-based theoretical studies and algorithms for autonomous flight have been presented. Among them, studies that enable drones to safely reach their destination without colliding with obstacles when performing specific tasks are drawing attention. These studies analyze the sensor data mounted on the drone and the surrounding data collected by the drone based on various machine learning algorithms.

In order for these technologies to be commercialized and used in a real life, verification and improvement through simulation of these studies are essential. Specifically, to verify autonomous flight technologies, a more realistic simulation platform that reflects the actual drone flight environment in real time is essential beyond simple theoretical verification. Furthermore, training UAVs in the real world is very challenging because UAVs are easily lost and damaged during flight, not to mentioning the training time and cost in the real world.

Thus far, many robot simulators [33,34,35] and simple machine learning test tools [43] have been developed. However, with solely using the existing robot simulators or simplified machine learning visualizers, we can test only robot motion operations or machine-learning algorithms but not consider both.To overcome this limitation, this paper proposes a holistic UAV simulation platform by integrating robot simulators and ML-based path planning algorithms.

The proposed platform creates physical environments virtually by linking existing flight simulators such as Gazebo and V-REP with machine learning engines. In particular, drones operate virtually in simulation and use sensors such as Lidar attached to these virtual drones to create map data for the virtual environment. Then, the simulator ROS node transfers the collected information to the HVIN ROS agent so that it can use the information as learning data for machine learning engines. The machine learning engine delivers the path planning results for the current state to the flight simulator output, which results in the drone having autonomous flight control in the simulator.

**Figure 9:**
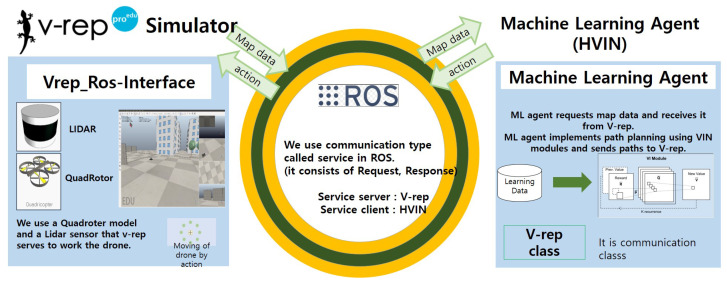
The proposed training and evaluation platform.

In the proposed platform shown in Figure 9, we use Vrep, a robot simulator that makes it easy to construct and deploy maps through GUI. In addition, a quadrotor and a Lidar sensor, VelodyneVPL_16, which is a drone model provided in the simulator, are adopted. Process communication between the proposed HVIN agent and Vrep is implemented through ROS. By defining a service type of ROS, they can exchange map data, drone location, target location, and action value, as shown in Figure 9.

### 4.2. Experiment Setup

To evaluate the performance of the proposed HVIN algorithms, we conducted experiments with the simulation platform described in Section 4.1. The drone was controlled by the *Vrep* simulator according to the “action value” received from a HVIN agent in a machine learning module. The action value from the HVIN agent was one of eight types according to the direction of the drone’s further progression.

Our experiments included HVIN training as well as autonomous flight tests. The basic training parameters of the original VIN model contained in the proposed HVIN are shown in Table 1. The training was conducted by randomly constructing a 16 m × 16 m grid image map (i.e., a local map) with random starting and arriving points.

In the training stage, HVIN agent receives a map with obstacles and free space from Vrep through ROS as an $input_{1}$. Then, it uses reward values generated from HVIN agent itself as $input_{2}$. Using these values, the agent performs a training procedure inside the VIN agent. The VI module in a VIN agent is a neural network that enables differentiable planning calculations. Each iteration in VI can be seen as transmitting the previous value function and the reward function through the convolution layer and the max-pooling layer. As an analogy, each channel of a convolution layer can be thought of as corresponding to the *Q*-function of a specific action, and the convolution kernel weight can be thought of as corresponding to the discounted transition probability. Therefore, by repeatedly applying the convolution layer *K* times, the same effect as the VI iteration *K* times is achieved. This concept is represented in detail in [20].

After training, we tested an autonomous flight for several local maps and obtained the success rate to reach the goal avoiding obstacles. The accuracy of the trained model for a local map is around 92.00%.

**Figure 10:**
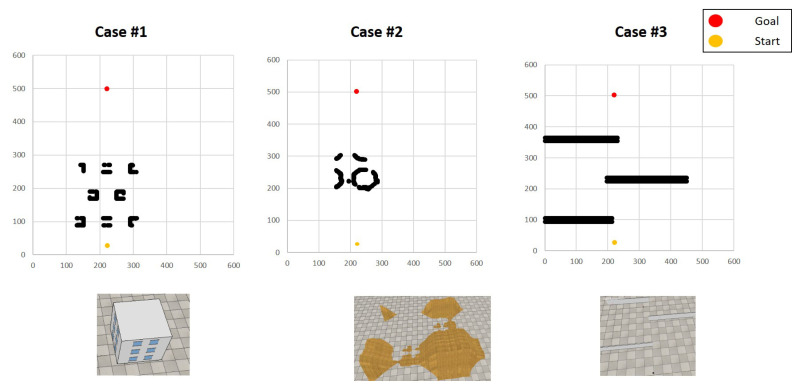
Evaluation Scenarios.

With the trained local area agent, we tested Multi-Layer and Fisheye HVIN algorithms for a wide target area. Using the proposed simulation platform, we designed three flight scenarios to evaluate the proposed algorithms. Figure 10 illustrates these three scenarios constructed in the experiments.

- **Case 1**: Arranging multiple buildings at intervals of 60 m
- **Case 2**: Placing large hills in the middle of the map
- **Case 3**: Placing long obstacles such as walls at intervals of 130 m

### 4.3. Evaluation Results

We tested the proposed Fisheye HVIN compared to Multi-Layer HVIN for all of the above scenarios with random start and end points. The objective of the proposed system was to get a drone to the final destination by avoiding collision. From this point of view, the flight was considered “failed” if the drone hit an obstacle during flight or did not progress for more than 30 s. Table 2 shows the results when we set up a starting point to (220, 25) and a target point to (220, 500) for all of the above scenarios.

From the results, we first observed that Fisheye HVIN is more advantageous to find the route and reach the destination than Multi-Layer HVIN in all three cases. This is because Fisheye HVIN contains more detailed area information around the drone, thus it can detour around obstacles better. In addition, Fisheye HVIN has a big picture about the entire map and so it can effectively find a path to a destination. On the other hand, a VIN agent of each layer in the Multilayer HVIN algorithm relies on a local view and heavily compressed information as a layer depth increases. Due to this feature, the success rate of Fisheye HVIN is higher than that of Multi-Layer HVIN in all cases. In addition, we noticed that the success rate of Multi-Layer HVIN of reaching the target point differed in the three cases. **Case 1** showed a relatively higher success rate than **Cases 2** and **3**. By analyzing flight simulation logs of these three cases, In **Cases 2** and **3**, there exist large obstacles spanning several local sections. Due to the spanned large obstacles, Multi-Layer HVIN with lack of global views might suffer more failure to find a proper route.

**Figure 11:**
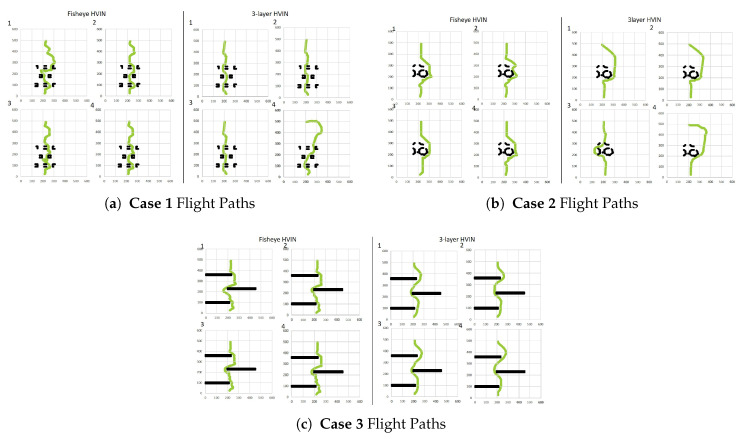
Simulation results.

We also performed in-depth analysis for each trial of path finding of these two algorithms. Generally speaking, the resulting flight paths were slightly different in all three scenarios, but there is no significant difference in flight paths among trials in each scenario, as shown in Figure 11. However, in Multi-Layer HVIN, sometimes the destination is reached by an inefficient route rather than an optimal route. The cause is that the drone might not be able to move right away in the direction of the action order by HVIN agents due to moment of physical inertia. Multi-Layer HVIN is affected more heavily by such phenomenon because Multi-Layer HVIN agent of each layer is constructed statically and also detailed information of each layer is hidden and transferred after compression to the upper layers, which causes information loss.

**Figure 12:**
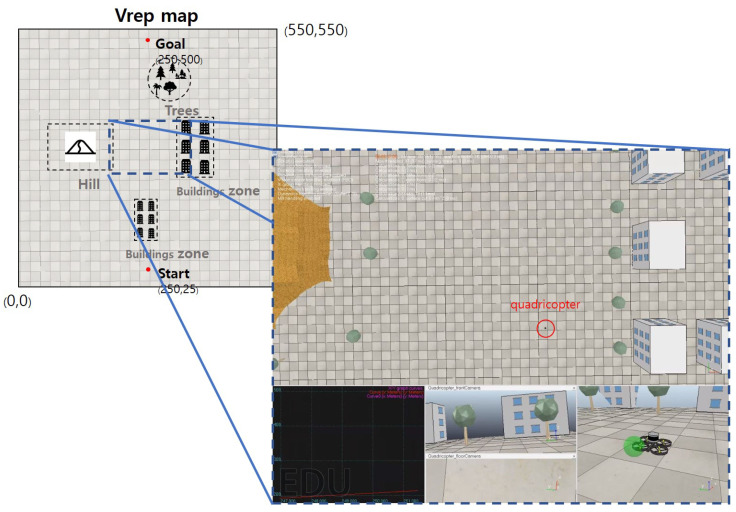
Realistic Experimental Setup.

To analyze the performance of Fisheye HVIN in detail, we conducted an additional experiment. In this experiment, we set up a more realistic simulation environment, as shown in Figure 12. The total target area was 550 m × 550 m (the maximum target area supported in Vrep is 600 m × 600 m) and we deployed two clusters of buildings with different heights, hills, and trees. The start and end points were (250, 25) and (250, 500), respectively, as presented on the left of Figure 12. The right of Figure 12 is a screenshot of a Vrep simulator around a drone. It shows the bird-eye view (in the top section), a camera view seen from the drone (in the middle picture of the bottom section), and a picture around the drone. With this setup, we tested autonomous flight path finding 10 times and measured the success ratio and the flight time from the source to destination. The quadrotor successfully found the way from the source to destination 10 out of 10 times. Table 3 shows the flight time to reach the end point in each trial as well as success or failure for each run. We observed that the drone reached the destination about 20 min in average. Considering the speed of the drone was set to 0.5~1 m/s and the target area size was 550 m × 550 m in our experiments, 20 min for path finding is reasonable because even a direct flight in a free space without an obstacle would take about 13–15 min to reach the goal on average.

Overall, we compared two HVIN agents based on the several experimental results and summarized main features, as shown in Table 4. From CPU usage point of view, Fisheye HVIN is more efficient than Multi-Layer HVIN. Fisheye HVIN is performed by up to two layers (only at local and global layers) regardless of the target area size. In contrast, Multi-Layer HVIN requires more layers as the size of the area to be applied for autonomous flight increases. Depending on the number of layers, the amount of data exchanged between Vrep and the HVIN agent is increased, and the operation procedure becomes complicated. As a consequence, a Multi-Layer HVIN consumes more CPU usage than a Fisheye HVIN agent.

In terms of data communication, Fisheye HVIN exchanges the drone’s action value, target and drone location, and local and global map data between two nodes. However, in the case of Multi-Layer HVIN, additional map data are needed depending on the number of layers so it requires more communication between a Vrep simulator and a HVIN agent. As for the size of the area that can be applied to autonomous flight, Fisheye HVIN can flexibly cover the area without limiting the size by controlling compression ratio (i.e., magnification). In contrast, Multi-Layer HVIN should add more layers as the target size increases because the number of sections at each layer is statically fixed to 16 × 16 in each layer. This might cause size limitation of Multi-Layer HVIN to the real target world.

In addition, to analyze the proposed Fisheye and Multi-layer HVIN algorithms, we also tested DQN (Deep Q Networks) [9,10]. DQN is one of the widely-used machine-learning-based path planning algorithms relying on a reinforcement learning model. With DQN, path planning was trained to create a path to the target point. Table 5 shows the training parameters of the DQN-based path planning simulations. Note that DQN is model-free and thus a DQN agent is trained through episodes in one environment. Once the training is done, UAVs can find their way to the destination in the trained environment. In other words, the “knowledge” trained with one environment is not well suited for the new environment. To overcome this feature, DQN trains the agent with a large number of episodes with **experience replay.** All transitions $<s,a,r,s^{\prime }>$ obtained during the training are stored in the replay memory. When collecting samples, it does not select the latest transition, but randomly selects samples of a batch size among the transitions stored in the replay memory. Because the transition obtained in various situations is used as a sample, a *Q*-value that is not biased to a special situation can be obtained.

With this feature, DQN is trained through a large number of episodes. Generally, the number of episodes of DQN training is about 10,000, whereas the number of episodes in VIN is about 30. To confirm this argument, we extended and modified the original DQN to accumulate the “knowledge” with multiple environments.

Unlike the proposed Fisheye HVIN, when using DQN-based autonomous flight, there is no case where the drone succeeded in reaching the target point in all of **Cases 1–3**.

**Figure 13:**
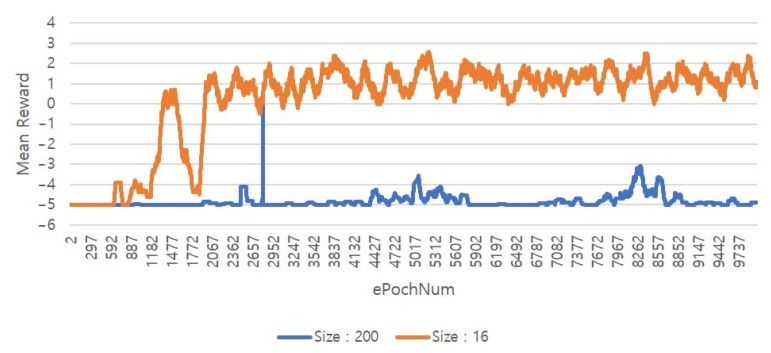
Reward values of a DQN algorithm.

To find the cause of such failure, we trained a DQN algorithm with varying the size of the autonomous flight area from 16 m × 16 m to 200 m × 200 grid area. We also set up a map as in **Case 1**. During training a DQN agent in 16 m × 16 m grid area, we observed that mean reward value per epoch is gradually increased. However, when the size of the map data increased to 200 m × 200 m, it was confirmed that learning itself is not effective, as presented in Figure 13. This result implies that DQN is not effective in a large area path planning.

**Figure 14:**
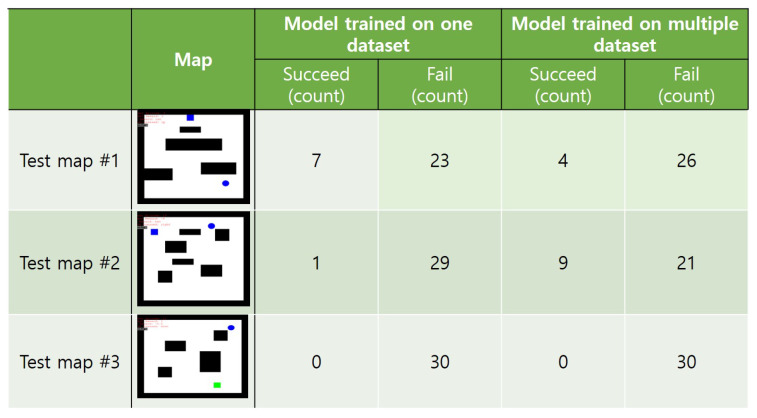
Evaluation results of a DQN algorithm.

Based on the training results, we tested DQN only in 16 m × 16 m grid and obtained the results shown in Figure 14. We trained a DQN agent with 10,000 epochs in two training scenarios: (1) only in one training environment; and (2) in five different environments with accumulating the trained “knowledge”. After that, we tested the trained DQN with three new maps represented in the leftmost column of Figure 14. From these results, we observed that training a DQN agent in multiple environments does not improve path finding accuracy.

In summary, we confirmed that, when using DQN, training becomes very inefficient when the size of the area applicable to autonomous flight increases. In addition, DQN does not perform well for path planning in new environments other than the trained environment.

## 5. Conclusions

In this study, Fisheye HVIN (Hierarchical VIN) was devised to utilize a reinforcement learning model for unmanned autonomous flight system. Specifically, we designed a Fisheye HVIN algorithm to apply different and adaptive map compression degree according to the location of drone where as Multi-Layer HVIN increases the number of VIN layers with the static compression rate as a target size increases.

To verify the Fisheye HVIN in a realistic way, we also developed a simulation platform integrating Vrep robot simulator and a machine learning HVIN agent through ROS. The developed simulation platform reflects a real environment considering physical features of UAV movement such as moment of inertia. The proposed Fisheye HVIN turned out to be an effective and efficient deep learning-based path planning algorithm even for a wide target area. Compared to the previous ML-based path planning algorithms, the proposed technique can be applied to wide target areas without sacrificing accuracy and speed. In addition, unlike DQN, the proposed Fisheye HVIN performs well for path planning in new environments other than the trained environment.

## Figures and Tables

**Table 1:** HVIN Training Parameters.

Parameter	Value
Image size	16
Number of value iterations	20
Number of channels in each input layer	2
Number of channels in the first convolution layer	150
Number of channels in q function convolution layer	10
Learning rate	0.002
Number of epochs to train	30
Batch size	128
Number of dataset	456,309

**Table 2:** Success ratio of “goal reached”.

Test Case	Proposed Algorithms	No. of Success Trials	No. of Failed Trials
**Case 1**	Fisheye HVIN	19	1
	Multi-Layer HVIN	17	3
**Case 2**	Fisheye HVIN	20	0
	Multi-Layer HVIN	7	13
**Case 3**	Fisheye HVIN	14	6
	Multi-Layer HVIN	9	11

**Table 3:** Success rate and flight time to reach a goal of Fisheye HVIN.

Each Trial	Success/Fail	Fight Time (min)
1	Success	20.1
2	Success	20.7
3	Success	19.6
4	Success	20.2
5	Success	21.6
6	Success	21.3
7	Success	20.6
8	Success	20.6
9	Success	19.9
10	Success	20.4

**Table 4:** Fisheye HVIN vs. Multi-Layer HVIN.

	Fisheye HVIN	Multi-Layer HVIN
CPU Usage	Planning is performed only in two layers, local and global.	As the number of layers increases, the number of HVIN agents running operations increases
Data Communication	Drone action, position, goal, local & global map data	Drone action, position, goal, map data as many as layers
Size of Target Area	Can support without limiting size by adjusting compression rate	Maximum size is limited by the number of layers
Simulator Overhead	Compression overhead in a simulator	Overhead for hierarchical map configuration

**Table 5:** DQN training parameters.

Parameter	Value
Image size	16 or 200
epsilon	0.1
Number of epochs to train	10,000
Learning rate	0.0001
Batch size	10

## Abbreviations

The following abbreviations are used in this manuscript:

UAV	Unmmaned Aerial Vehicle
DQN	Deep Q Networks
VIN	Value Iteration Networks
HVIN	Hierarchical Value Iteration Networks
ML	Machine Learning
ROS	Robot Operating System
DDPG	Deep deterministic policy gradient algorithm
AMP	Autonomous motion planning
